# Impacts of plasma microbial lipopolysaccharide translocation on B cell perturbations and anti-CD4 autoantibody production in people with HIV on suppressive antiretroviral therapy

**DOI:** 10.1186/s13578-023-01022-6

**Published:** 2023-05-03

**Authors:** Xiaoyu Fu, Da Cheng, Zhenwu Luo, Sonya L. Heath, Ruth Adekunle, John E McKinnon, Lisa Martin, Zizhang Sheng, Enrique Espinosa, Wei Jiang

**Affiliations:** 1 Department of Microbiology and Immunology, Medical University of South Carolina, 173 Ashley Ave. BSB207, Charleston, SC 29425 USA; 2 Department of Medicine, Division of Infectious Diseases, University of Alabama at Birmingham, Birmingham, AL 35294 USA; 3 Division of Infectious Diseases, Department of Medicine, Medical University of South Carolina, 173 Ashley Ave. BSB207, Charleston, SC 29425 USA; 4 Ralph H. Johnson VA Medical Center, Charleston, SC 29401 USA; 5 Aaron Diamond AIDS Research Center, Columbia University Vagelos College of Physicians and Surgeons, New York, NY 10032 USA; 6 Laboratory of Integrative Immunology, National Institute of Respiratory Diseases “Ismael Cosío Villegas”, Mexico City, 14080 Mexico

**Keywords:** HIV, ART, Anti-CD4 IgG, B cells, Microbial LPS translocation

## Abstract

**Background:**

. Up to 20% of people with HIV (PWH) who undergo virologically suppressed antiretroviral therapy (ART) fail to experience complete immune restoration. We recently reported that plasma anti-CD4 IgG (antiCD4IgG) autoantibodies from immune non-responders specifically deplete CD4 + T cells via antibody-dependent cytotoxicity. However, the mechanism of antiCD4IgG production remains unclear.

**Methods:**

. Blood samples were collected from 16 healthy individuals and 25 PWH on suppressive ART. IgG subclass, plasma lipopolysaccharide (LPS), and antiCD4IgG levels were measured by ELISA. Gene profiles in B cells were analyzed by microarray and quantitative PCR. Furthermore, a patient-derived antiCD4IgG–producing B cell line was generated and stimulated with LPS in vitro. B cell IgG class switch recombination (CSR) was evaluated in response to LPS in splenic B cells from C57/B6 mice in vitro.

**Results:**

. Increased plasma anti-CD4 IgGs in PWH were predominantly IgG1 and associated with increased plasma LPS levels as well as B cell expression of TLR2, TLR4, and MyD88 mRNA in vivo. Furthermore, LPS stimulation induced antiCD4IgG production in the antiCD4IgG B cell line in vitro. Finally, LPS promoted CSR in vitro.

**Conclusion:**

. Our findings suggest that persistent LPS translocation may promote anti-CD4 autoreactive B cell activation and antiCD4IgG production in PWH on ART, which may contribute to gradual CD4 + T cell depletion. This study suggests that reversing a compromised mucosal barrier could improve ART outcomes in PWH who fail to experience complete immune restoration.

**Supplementary Information:**

The online version contains supplementary material available at 10.1186/s13578-023-01022-6.

## Introduction

Antiretroviral therapy (ART) is an effective treatment for people with HIV (PWH) that generally results in viral suppression, increased CD4 + T cell counts, and improved immune function. However, approximately 20% of patients on ART do not achieve complete immune restoration, exhibiting persistently low CD4 + T cell counts despite long-term viral suppression; these patients are referred to as immunological non-responders (INRs) [1–3]. Compared with immune responders (IRs), INRs have a significantly increased incidence of morbidity and mortality from AIDS-related and non–AIDS-related diseases [4–6].

Various factors have been implicated in the incomplete immune restoration seen in INRs, such as older age, delayed ART initiation, low nadir and baseline CD4 + T cell counts, impaired bone marrow hematopoietic or thymic function, reduced thymic output, residual viral replication, abnormal immune activation and inflammation, and lymphoid tissue fibrosis [7–12]. Recently, several studies reported the role of autoantibodies in infectious disease severity [13–15], and autoimmunity is recognized for its pathogenic impact on infectious diseases in the absence of any clinical autoimmune disease. We previously showed that anti-CD4 IgG (antiCD4IgG) levels were significantly increased in INRs compared with both IRs and healthy volunteers [16]. Furthermore, patient-derived antiCD4IgG induced antibody-dependent cell-mediated cytotoxicity (ADCC) resulting in CD4 + T cell apoptosis in vitro [17], suggesting that inappropriate production of autoreactive IgG results in depletion of CD4 + T cells in INRs. More recently, the effects of anti-CD4 autoantibodies on CD4 + T cell depletion in HIV were revealed by A Lisco, C-S Wong, SL Lage, I Levy, J Brophy, J Lennox, M Manion, MV Anderson, Y Mejia, C Grivas, et al. [18]. Nevertheless, the driver for antiCD4IgG remains unclear.

The administration of bacterial lipopolysaccharide (LPS) can induce autoantibodies and autoimmunity in animals [19, 20]. Thus, we hypothesize that increased B cell activation and autoantibody production is mediated by elevated microbial products in the bloodstream via the impaired gut mucosal integrity in HIV. Consistently, plasma LPS remain elevated in INRs compared with healthy individuals [21]; elevated plasma levels of LPS and enterobacterial DNA are associated with immune nonresponse in PWH on ART [22]; plasma levels of soluble CD14, a marker of microbial translocation, are higher in PWH on ART than in healthy controls [23]; and plasma levels of bacterial LPS are significantly higher in PWH compared with healthy controls and correlate with a less effective response to ART, as indicated by higher T cell activation and decreased CD4 + T cell counts [24]. However, despite the apparent correlation between markers of microbial translocation in the bloodstream and the failure of ART to restore CD4 + T cell counts, the mechanism underlying this relationship remains unclear. Thus, the present study aimed to determine whether the presence of microbial products in the bloodstream directly stimulates anti-CD4 autoantibody production by B cells in PWH who are undergoing ART.

## Materials and methods

### Human subjects

The study participants included 16 healthy individuals and 25 PWH on suppressive ART for at least 2 years. The inclusion criteria were as follows: (1) men and women aged 18 or older, (2) able and willing to provide informed consent, (3) healthy individuals who self-reported as being HIV-negative, and (4) PWH on ART with a plasma HIV RNA below the limit of detection for at least 24 weeks (a single reading above 500 copies/mL was allowed). The exclusion criteria were as follows: (1) self-reported pregnancy or breast-feeding, (2) recent severe illness such as anemia, (3) need for or use of specific medications (such as antibiotics, systemic immunomodulatory agents, and supraphysiologic doses of steroids (> 10 mg/day)) during the 120 days before enrollment, and (4) any other condition that rendered a person unsuitable for the study or unable to comply with study requirements, as determined by the lead investigator. This study was approved by the Medical University of South Carolina (Pro00020606). All study participants provided signed informed consent. All PWH had at least 2 years of undetectable plasma HIV-1 RNA. The background clinical features of the participants are summarized in Table 1. A cutoff value of 50 ng/mL antiCD4IgG was used to distinguish between high versus low antiCD4IgG groups because 95% of the healthy participants had plasma anti-CD4 IgG levels lower than this value. Thus, PWH on ART were stratified into low and high autoCD4IgG subgroups.

**Table 1:** Clinical characteristics of study subjects

	HIV-negative (n = 16)	HIV+/antiCD4low (n = 17)	HIV+/antiCD4high (n = 8)	P value (two HIV + groups)
Age (years)	45 (37–57)	48 (38–57)	44 (37–59)	0.18
Sex ratio, male: female	7:9	12:5	5:3	0.52
CD4^+^ T cell counts		568 (359–735)	278 (96–348)	< 0.0001
Nadir CD4^+^ T cell counts		294 (157–510)	215 (101–425)	0.47
Duration of ART (years)		6.2 (4.3–6.9)	5.5 (4–6)	0.56

ART, antiretroviral therapy; CD4 T cell count (cells/µL)

Data are means (25th percentile, 75th percentile)

### Enzyme-linked immunosorbent assay

Microtiter plates were coated with either 1 µg/100 µL/well soluble CD4 protein (sCD4, Progenics Tarrytown, NY) or a 1:20 dilution of the 2013–2014 seasonal influenza antigens at 4 °C overnight. After washing, diluted plasma was added to each well and allowed to incubate for 1 h at room temperature. After washing, horseradish peroxidase–labeled goat anti-human IgG, IgM, IgA (KPL, Gaithersburg, MD), or IgG1-4 subtypes (Thermo Fisher Scientific, Waltham, MA) were added to the microtiter wells and incubated at room temperature with gentle shaking for 60 min. Next, the 2,2’-Azino-di(3-ethylbenzthiazoline-6-sulfonate) substrate solution was added to detect binding. Absorbance was measured at 405 nm. All measurements were performed in duplicate, and the values were normalized to a standard curve generated using the human monoclonal anti-CD4 antibody zanolimumab (HuMax-CD4; Genmab) (0.05–10 ng/mL).

### Quantitative PCR (qPCR) analysis and microarray of mRNA expression in B cells

B cells were isolated using a negative selection kit (Qiagen, Hilden, Germany). The purity of the isolated cells was always above 96%. TRizol (MRC, UK) was used to isolate total RNA, and cDNA was synthesized by reverse transcription using M-MLV reverse transcriptase (Promega Corporation, Madison, WI, USA) and random primers. The obtained cDNA was diluted at 1:25 with water, and 10 µL of the diluted solutions were used as the template for the amplification step. Parameter-specific primer sets optimized for the LightCycler (RAS) were developed by and purchased from Search-LC (Heidelberg, Germany); the primer sequences were described previously [25]. The PCR reaction was performed using a LightCycler FastStart DNA SYBR Green I kit (RAS), with conditions specified by the primer manufacturer. The method of microarray analysis was described in our previous studies [26]. Briefly, total RNA was extracted from 2 × 10^6^ B cells using the RNeasy Micro kit (Qiagen), quantitated, qualified with Agilent 2100 Bioanalyzer (Agilent Technologies, Santa Clara, CA), and run with Affymetrix GeneChip assays. The Affymetrix Human GeneChip U133 Plus 2.0 Array (Affymetrix, Santa Clara, CA) was used for RNA hybridization and labeling according to the manufacturer’s protocol. The analysis of the scanned images and signal values for each probe set was obtained with the GCOS (Affymetrix). R program (3.3.1) was used for microarray data analysis.

### Measurement of plasma LPS levels

Plasma samples were diluted to 10% in endotoxin-free water and heated at 80 °C for 10 min to inactivate inhibitory plasma proteins. The LPS levels in the diluted plasma samples were then quantified using a Limulus amebocyte lysate QCL-1000 kit (Lonza), as described previously [27].

### Generation of a patient-derived anti-CD4 IgG–producing B cell line

PBMCs were obtained from an INR participant who expressed high levels of anti-CD4 IgG by density gradient centrifugation of whole heparinized blood. Sorted B cells were transformed in vitro by culturing in EBV-containing supernatants collected from the B95-8 cell line (ATCC® VR-1492™). The transformed B cells were then seeded at approximately 1000 cells/well in 384-well plates in complete medium containing 3 mg/mL CpG2006 (InvivoGen). After 2 weeks, the culture supernatants were screened for specific antibody production by ELISA. Positive cultures were cloned by limiting dilution in the presence of CpG 2006 and irradiated mononuclear cells.

### In vitro B cell stimulation

The patient-derived anti-CD4 B cell line was cultured in complete RPMI medium, consisting of RPMI-1640 supplemented with 15% fetal bovine serum (FBS, vol/vol), 50 µg/mL penicillin/streptomycin, and 1 mM sodium pyruvate. LPS (50 ng/mL, *E. coli* O55:B5, InvivoGen, San Diego, CA), inactivated *Staphylococcus aureus*, *Pseudomonas aeruginosa*, cytomegalovirus (10^7^ CFU/mL), IL-23, or IL-17 (50 ng/mL) was used to stimulate cells for 10 days. Cell culture supernatants were collected for evaluation of cell counts and anti-CD4 IgG production.

## B cell class switch DNA recombination (CSR)

B cells were isolated from the spleen of 8- to 10-week-old unmanipulated C57BL/6J mice using the B Cell Negative Isolation Kit (STEMCELL Technologies, Cambridge, MA). B cells were cultured with complete medium and 50 µM of β-mercaptoethanol (Thermo Fisher Scientific) at 5 × 10^5^ cells/mL then treated for 96 h with medium, LPS (3 µg/mL) plus IL-4 (5 ng/mL, Biolegend), IFN-γ (50 ng/mL, BioLegend] or TGF-β1 (2 ng/mL, R&D Systems, Minneapolis, MN). Cells were harvested and stained with Ghost Dye™ Red 780 (TONBO, San Diego, CA). CSR was analyzed by the percentages of proliferating IgG3 and IgG2b cells using a BD FACSVerse™ Cell Analyzer (BD, San Jose, CA) after surface staining with anti-B220 (RA3-6B2), anti-IgG2b (RMG2b-1), anti-IgG3 (R40-82), anti-CD138 (281-2), and anti-IgM (RMM-1) from BioLegend.

### Statistical analyses

The non-parametric Mann-Whitney test between two groups and one-way ANOVA with the Kruskal-Wallis test for more than two groups were used to determine differences in continuous measurements. Spearman’s correlation test was used to determine associations. The data were analyzed using GraphPad Prism software Version 8.3.0 (San Diego, CA, USA) and R program (3.3.1); P < 0.05 was considered statistically significant.

## Results

### Anti-CD4 IgG1 is the predominant subclass in PWH on ART

**Fig. 1:**
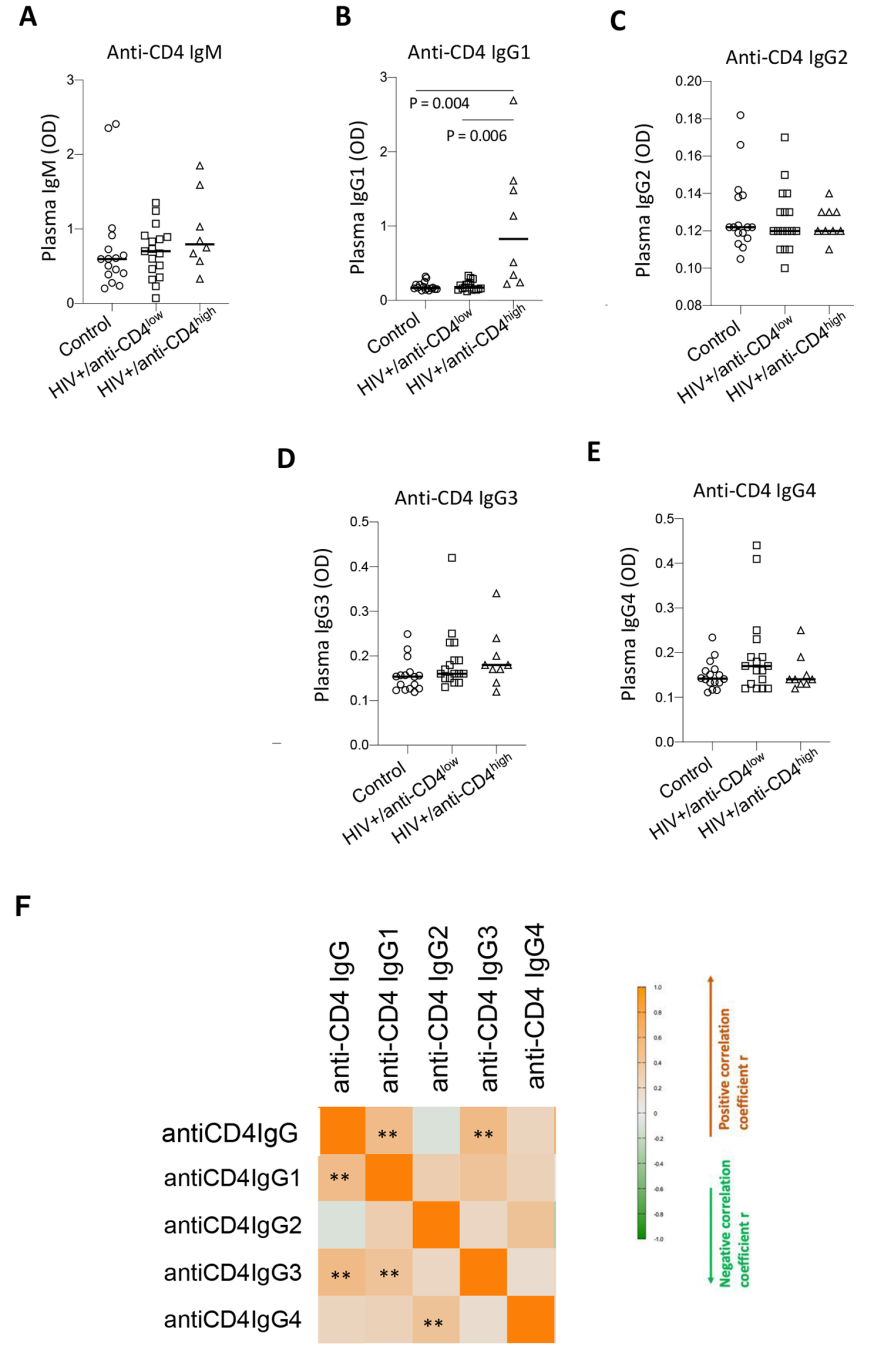
Anti-CD4 IgG1 is the predominant subclass in PWH on ART. Plasma levels of anti-CD4 IgM (**A**), IgG1 (**B**), IgG2 (**C**), IgG3 (**D**), and IgG4 (**E**) were assessed by ELISA from healthy controls (n = 16), and PWH on ART with high (HIV+/antiCD4^high^, n = 8) versus low (HIV+/antiCD4^low^, n = 17) antiCD4IgG levels. ANOVA tests were used to analyze data. **(F)** Correlations between antiCD4IgG and subclasses. Spearman correlation tests. *, P < 0.05; **, P < 0.01; ****, P < 0.0001

IgG1 and IgG3 antibodies can induce ADCC, unlike IgG2 and IgG4 [28]. We first investigated anti-CD4 IgM and IgG subclasses in PWH on ART. To address this, we performed an ELISA of plasma samples from all study participants including healthy controls and PWH with high antiCD4IgG or low antiCD4IgG. As expected, anti-CD4 IgM levels were similar in healthy controls and all PWH on ART, regardless of respective anti-CD4 IgG levels (Fig. 1A). In contrast, anti-CD4 IgG1 was the predominant subclass in PWH on ART with high antiCD4IgG levels compared to PWH on ART with low antiCD4IgG levels, while all other subclasses were expressed at similar levels in all groups (Fig. 1B–D). Correlation analysis indicated a significant relationship between antiCD4IgG and anti-CD4 IgG1, as well as, unexpectedly, anti-CD4 IgG3. These results suggest that anti-CD4 IgG1 may be primarily responsible for the effects of anti-CD4 autoantibodies observed in PWH on ART.

### High levels of antiCD4IgGs are associated with increased plasma LPS levels and B-cell TLR4 (not TLR2, TLR7, or TLR9) mRNA expression, and B-cell gene profiles related to microbial translocation

**Fig. 2:**
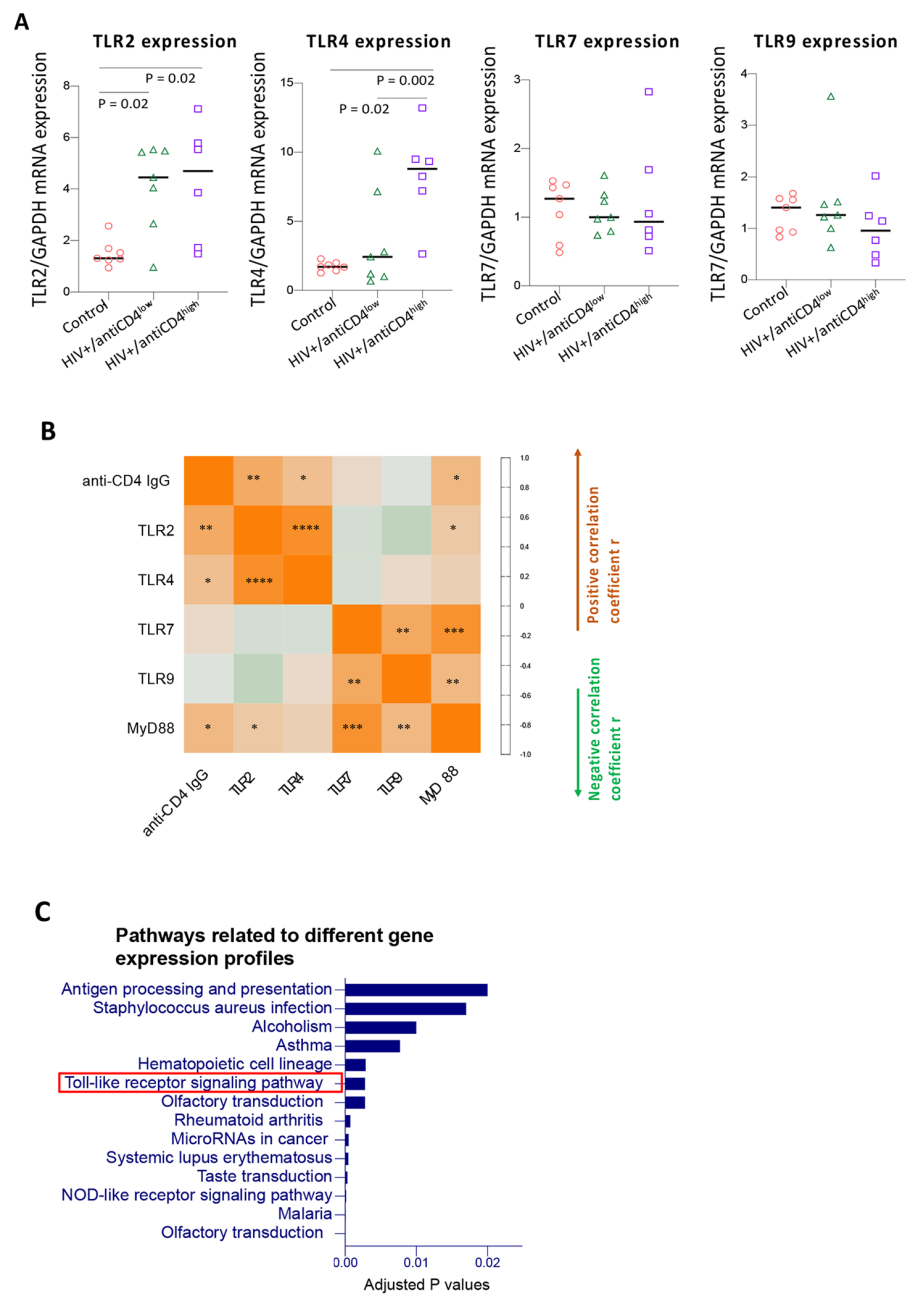
High plasma levels of antiCD4IgG are associated with increased TLR2 and TLR4 expression and distinct cell signaling pathway activation in B cells from PWH on ART. Total mRNA was extracted from B cells isolated from healthy controls (n = 7) and PWH on ART with high (HIV+/antiCD4^high^, n = 6) versus low (HIV+/antiCD4^low^, n = 7) anti-CD4 IgG levels. (**A**) Relative mRNA expression of TLR2, TLR4, TLR7, and TLR9 versus GAPDH in B cells as determined by qPCR. ANOVA tests were used to analyze data. High: antiCD4IgG > 50 ng/mL; Low: antiCD4IgG ≤ 50 ng/mL. (**B**) Correlations between plasma anti-CD4 autoantibody levels and TLR and MyD88 mRNA expression in B cells. *, P < 0.05; **, P < 0.01; ****, P < 0.0001. **(C)** The representative immunological pathways were enriched in coherently changed genes between the two HIV groups

Our previous study showed the link between plasma microbial translocation and autoantibody levels in systemic lupus erythematosus (SLE) autoimmune disease [29]. We speculated that characteristics of B cell gene expression in PWH on ART who present with high levels of antiCD4IgGs may reflect gene profiles related to long-term persistent microbial product translocation in the bloodstream. Therefore, we assessed B cell gene expression profiles by analyzing the mRNA expression of TLR signaling molecules in B cells. We found that PWH on ART with high plasma antiCD4IgG levels had increased TLR4 but similar levels of TLR2, MyD88, TLR7, and TLR9 mRNA expression in B cells compared with PWH on ART with low plasma antiCD4IgG levels (Fig. 2A, Supplementary Fig. 1A). Intriguingly, PWH on ART with low plasma antiCD4IgG levels also exhibited elevated TLR2 expression, but not TLR4 expression, compared with the healthy controls (Fig. 2A). Furthermore, correlation analysis showed that plasma antiCD4IgG levels correlated with IgG1 and IgG3 subclasses, as well as with levels of TLR2, TLR4, and MyD88 mRNA expression in B cells (Fig. 2B). B cell gene microarray analysis showed that several pathways of gene profiles were associated with microbial translocation, autoimmunity, and others in PWH with high versus low antiCD4IgG levels (Fig. 2C). Taken together, these results suggest that anti-CD4 autoreactive B cell activation in PWH on ART likely involves microbial product–mediated signaling pathways such as TLR4 in vivo.

### Plasma LPS, a marker of microbial translocation, stimulates autoantibody production in an antiCD4IgG producing B cell line in vitro.

**Fig. 3:**
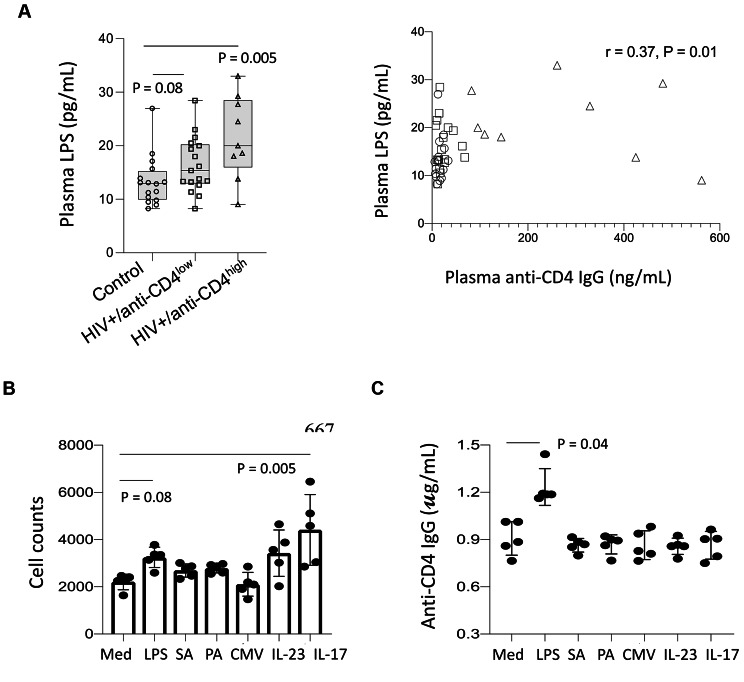
LPS correlated with antiCD4IgG production in vivo and stimulated antiCD4IgG production by B cells *in vitro.* Plasma LPS levels in healthy controls and virologically suppressed PWH on ART who presented with high versus low antiCD4IgG levels were evaluated by LAL assay. (**A**) Plasma LPS levels in the three study groups. Correlation between antiCD4IgG and LPS levels. B cell counts (**B**) and proliferation (**C**) in response to LPS, *S. aureus*, *P. aeruginosa*, CMV, IL-23, or IL-17 in a patient-derived antiCD4IgG-producing B cell line. ANOVA and Spearman correlation tests were used to analyze data

The elevated TLR2 and TLR4 expression observed in B cells from the high antiCD4IgG PWH group suggested innate immune activation via TLR2 and TLR4 in these patients in response to microbial products (e.g., bacterial LPS). Therefore, we evaluated plasma levels of LPS, a marker of systemic microbial translocation. Anti-influenza IgG was assessed as a control, as all subjects had received the seasonal influenza vaccine in the preceding year. We found that LPS levels increased in the PWH group with high antiCD4IgG levels versus healthy controls, and marginally increased in the high antiCD4IgG group versus the low antiCD4IgG group (Fig. 3A). There was a direct correlation between plasma LPS and antiCD4IgG levels in all subjects tested (Fig. 3A); however, the correlation was mainly driven by the subjects with high anti-CD4 IgG levels but not the subjects with low anti-CD4 IgG levels; furthermore, r = 0.37 is a weak correlation (Fig. 3A). This correlation was not detected between plasma LPS and anti-influenza IgG (Supplementary Fig. 1B). Given the observed correlation between the plasma LPS levels and antiCD4IgG levels, we investigated whether a causal relationship existed. To test this, we generated an antiCD4IgG–producing B cell line from one non-responder study participant and stimulated those cells with LPS, whole inactivated *S. aureus*, *P. aeruginosa*, CMV, IL-23, or IL-17 (two cytokines related to microbe-induced mucosal inflammation) for 10 days in vitro. Whole bacteria and CMV virus were used as controls. We found that stimulation with IL-17 significantly increased cell counts at the end of the culturing period, while stimulation with LPS tended to increase cell counts but did not achieve statistical significance (Fig. 3B). Furthermore, LPS increased antiCD4IgG production, unlike any of the other stimuli (Fig. 3C). Taken together, these findings suggest that elevated IL-17 levels associated with mucosal inflammation promote B cell proliferation, and LPS may contribute to autoreactive B cell activation and production of antiCD4IgG in PWH on ART.

### LPS drives CSR

**Fig. 4:**
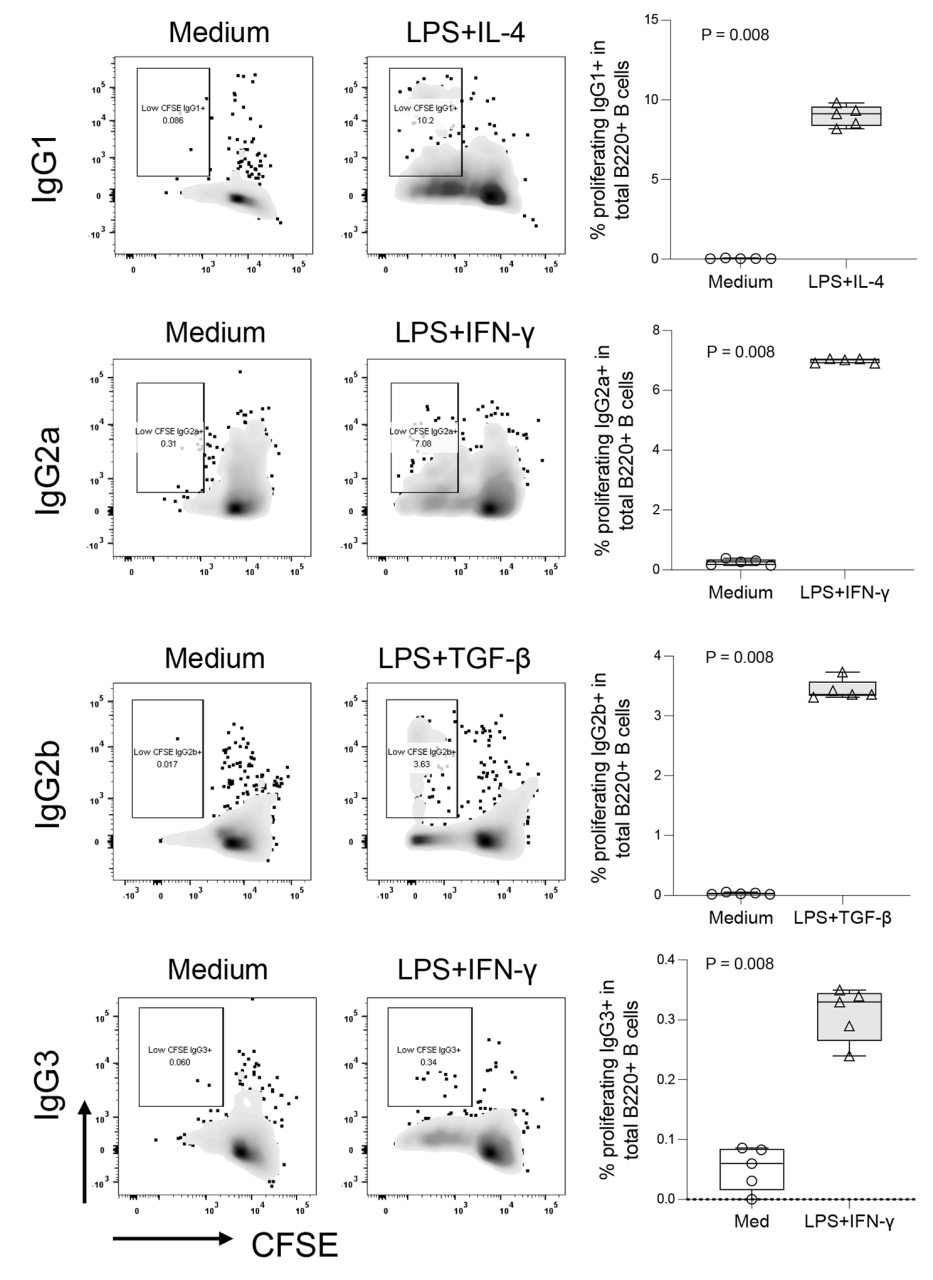
LPS promotes IgG class switch recombination in vitro. B cells were isolated from C57BL/6J mice and cultured with medium, LPS (3 µg/mL) plus IL-4 (5 ng/mL), IFN-γ (50 ng/mL), or TGF-β1 (2 ng/mL) for 96 h. Shown are the representative dot plots and summarized percentages of proliferating IgG subclass B cells in total B cells (%CFSE^low^IgG1/2a/2b/IgG3 + in B220 + cells). Non-parametric Mann-Whitney tests were used to analyze data

CSR is a crucial biological process for antibody maturation in response to both foreign antigens and self-antigens in the germinal center [30]. To investigate the mechanism of LPS-mediated autoantibody production, we culture mouse splenic B cells with LPS and cytokines related to CSR in mice. B cells were isolated from C57BL/6J mice and cultured with medium, LPS (3 µg/mL) plus IL-4 (5 ng/mL), IFN-γ (50 ng/mL), or TGF-β1 (2 ng/mL) for 96 h. The percentages of proliferating IgG subclass B cells in total B cells (%CFSE^low^IgG1/2a/2b/IgG3 + in B220 + cells) were evaluated for CSR. LPS plus cytokines promoted IgG CSR to IgG1, 2a, 2b, and 3, but LPS alone had the limited ability to drive CSR in vitro (Fig. 4 and Supplementary Fig. 2). These results are consistent with previous studies [31, 32].

## Discussion

**Fig. 5:**
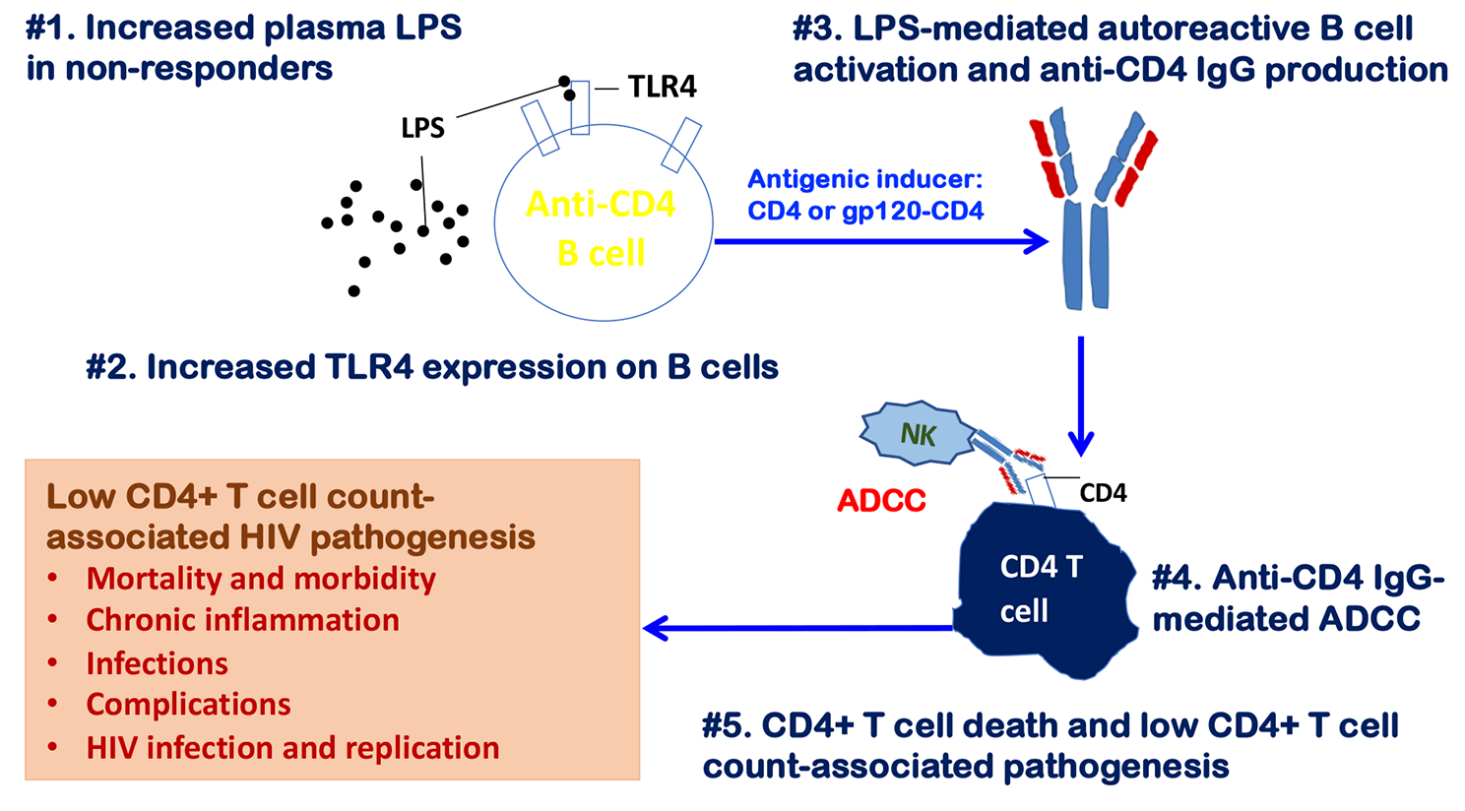
A model is proposed for increased plasma microbial product translocation–mediated B cell activation and anti-CD4 pathogenic autoantibody production leading to decreased CD4 + T cell counts in virologically suppressed PWH on ART. In our model, elevated plasma levels of LPS due to impaired mucosal barriers repeatedly stimulated the immune system resulting in increased TLR2 and TLR4 expression in B cells, with different one-time effects and high dose effects. The anti-CD4IgG–producing B cells then produced antiCD4IgGs in response to CD4 or HIV gp120-CD4. The resulting high plasma levels of antiCD4IgG induced CD4 + T cell death through ADCC, leading to the failure of immune reconstitution despite viral suppression with ART.

Autoimmunity plays a pathogenic role in infectious diseases in the absence of any clinical autoimmune disease [13–15]. In this study, we found that elevated plasma microbial LPS translocation was associated with B cell activation and antiCD4IgG production in PWH on ART. Furthermore, stimulation of LPS induced antiCD4IgG production in a patient-derived B cell line, and CSR in isolated splenic B cells from C57/B6 mice in vitro. Taken together, our findings suggest that a comprised mucosal barrier and systemic microbial product translocation may induce B cell activation and anti-CD4 autoantibody production in some PWH on ART, resulting in failure of immune restoration (Fig. 5).

Consistent with previously published studies, we found that plasma antiCD4IgG levels remained elevated in PWH on ART, even though levels of other HIV infection-associated autoantibodies decreased following ART [16, 33, 34]. HIV infection prior to ART is associated with polyclonal autoreactive B cell activation and the production of various IgG autoantibodies, including antiCD4IgG, suggesting nonfunctional autoantibodies with low affinity mediated by chronic inflammation [27, 35–38]. In contrast, antiCD4IgGs generated during suppressive ART can be pathogenic, mediate CD4 + T cell death via ADCC, and play a role in poor CD4 + T cell recovery after ART [16, 18, 39, 40]. Anti-CD4 IgG1 is the predominant subclass in PWH, which is consistent with its ADCC activity [16, 40].

While earlier studies have reported autoreactive B cell activation in untreated PWH, here we show activation of B cells in PWH on ART. The innate immune pathway activation is consistent with elevated plasma LPS observed in PWH on ART who have high levels of antiCD4IgG compared with healthy controls [41]. Similarly, RF Tanko, AP Soares, TL Müller, NJ Garrett, N Samsunder, Q Abdool Karim, SS Abdool Karim, C Riou and WA Burgers [42] reported that perturbations of B cell profiles largely normalized in PWH after 12 months on ART, but B cell activation remained high and was positively associated with plasma sCD14 levels [42]. Our findings show that this B cell activation specifically involves the upregulation of components of signaling pathways related to microbial products.

We further found that in vitro stimulation of LPS promoted antiCD4IgG production in patient-derived antiCD4IgG-producing B cells, likely due to increased TLR4 expression, a main receptor for LPS. This is unexpected, given that B cells from healthy humans generally do not express TLR4 and do not respond to LPS stimulation [43, 44]. In previous studies, short-term high dose of LPS stimulation may result in TLR4 downregulation in cells in vitro [45–47] but conflict results were also reported [48, 49]. Nonetheless, the effect of long-term low dose of LPS repeated stimulation on TLR4 remains unknown. This in vitro finding, in combination with the correlation that we observed between plasma LPS levels and antiCD4IgG levels in PWH on ART, implies that increased TLR4 expression may be functional and mainly on autoreactive B cells which result in the atypical autoreactive B cell activation and autoantibody production due to persistent microbial LPS translocation. Indeed, the increased TLR4 expression on CD8 + T cells has been found in patients with Rheumatoid Arthritis (RA) and responded to LPS to produce inflammatory cytokines; these CD8 + T cells contributed to inflammation and RA disease pathogenesis [50]. Besides autoimmunity, it remains unknown whether the increased TLR4 expression accounts for the LPS-sensitizing controls of infection.

In general, a “leaky” barrier and increased microbial product translocation (e.g., inflammatory bowel disease) associate with increased various autoantibodies in the absence of pathogenic activities [51, 52]. However, in individuals with a genetic predisposition or with immune perturbations (e.g., HIV), bacterial products (e.g., LPS) may enter the body via a leaky gut and trigger the development of pathogenic autoantibody production with or without clinical autoimmune diseases [53]. Moreover, the dose of LPS used the in vitro assay was much higher than those in the plasma. We assume that anti-CD4 IgG-producing B cells are activated and anti-CD4 IgGs are produced in the gut lymph nodes but not in the circulation in vivo. Thus, the LPS concentration is expected much higher in the gut lymph nodes compared to the blood. Thus, autoreactive B cells in the gut lymph nodes may be activated to produce autoantibodies in response to a high concentration of LPS. Finally, whether sorted antigen-specific B cells or IgG + or IgM + B cells respond to LPS directly deserves further investigations.

In conclusion, our study provides strong evidence that the mechanism underlying the failure of immune restoration in INRs involves microbial translocation into the bloodstream and, subsequently, LPS–mediated B cell activation and production of antiCD4IgGs that specifically deplete the CD4 + T cell population. This suggests that reversing a compromised mucosal barrier could improve ART outcomes in PWH who fail to experience complete immune restoration.

## Electronic Supplementary Material

Below is the link to the electronic supplementary material.

Supplementary Material 1

## Data Availability

Data are available upon request.

## Declarations

### Accession numbers

Microarray data are available in GEO (Gene Expression Omnibus, https://www.ncbi.nlm.nih.gov/geo/query/acc.cgi?acc=GSE109837) with the accession number GSE109837.

## Abbreviations

ADCC: antibody-dependent cell-mediated cytotoxicity
ART: antiretroviral therapy
INR: immunological nonresponder
IR: immune responder
PWH: people living with HIV
qPCR: quantitative PCR
