# Supplementary material for: Impacts of plasma microbial lipopolysaccharide translocation on B cell perturbations and anti-CD4 autoantibody production in people with HIV on suppressive antiretroviral therapy
Source: Cell Biosci. 2023 May 3;13:78. doi: 10.1186/s13578-023-01022-6 (PMC10157945; doi:10.1186/s13578-023-01022-6)
Supplement: Supplementary file 1 — Supplementary Material 1 [file 13578_2023_1022_MOESM1_ESM.docx]

P = NS

**Supplementary Figure 1**. Similar MyD88 mRNA expression in B cells occurred in the three study groups, with no correlation between plasma LPS and anti-influenza IgG levels. Distinct cell signaling pathway activation occurred in B cells from PWH on ART. Total mRNA was extracted from B cells isolated in PBMCs from the three study groups. (**A**) Relative mRNA expression of MyD88 versus GAPDH in B cells was determined by qPCR. ANOVA tests were used to analyze data. (**B**) No correlation occurred between plasma antiCD4IgG levels and MyD88 mRNA expression in B cells.

**Supplementary Figure 2**. IgG class switch recombination *in vitro*. B cells were isolated from C57BL/6J mice and cultured with medium, LPS (3 μg/mL), or LPS plus IL-4 (5 ng/mL), IFN-γ (50 ng/mL), or TGF-β1 (2 ng/mL) for 96 hours. The percentages of IgG subclass in total B cells in total B cells. Wilcoxon matched-pairs signed rank tests.
